# Supplementary material for: Pathophysiological Significance of Neutrophilic Transfer RNA-Derived Small RNAs in Asymptomatic Moyamoya Disease
Source: Cells. 2021 May 1;10(5):1086. doi: 10.3390/cells10051086 (PMC8147334; doi:10.3390/cells10051086)

2D Structure | **Local AU** | **Position**

```
238                          8mer-1a    267
 5'-agaAACCCTATGAGTGTAAGG-AATGTGGGa-3' utr
       ||  ||||   |  :|:  ||:  |||||||
 3'-uccUUAGGAU--UGGCGAUCUGGUACACCCu-5' tRF
                   16151413    7 6 5 4 3 2
                 3' Pairing      Seed
```

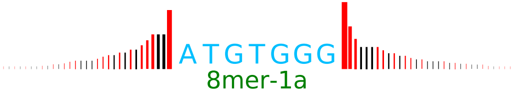

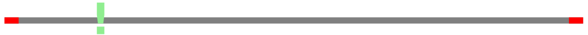

2D Structure | **Local AU** | **Position**

```
342                         8mer-1a    369
 5'-agaccTTATAAATGTAAGA-CATGTGGGa-3' utr
           |:  |||  :|:  |||  |||||||
 3'-uccuuAGGAUUGGCGAUCUGGUACACCCu-5' tRF
                 16151413    7 6 5 4 3 2
               3' Pairing      Seed
```

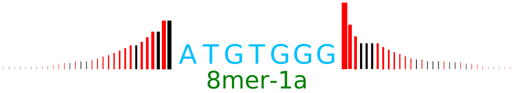

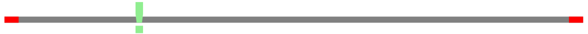

2D Structure | **Local AU** | **Position**

```
156                          8mer-1a    185
 5'-agaAACCCTATGAGTGTAAGCAATGTGGGa-3' utr
       ||  ||||     |:  ||  :|  |||||||
 3'-uccUUAGGAUUGGCG-AUCUGGUACACCCu-5' tRF
                 1615  1413    7 6 5 4 3 2
               3' Pairing      Seed
```

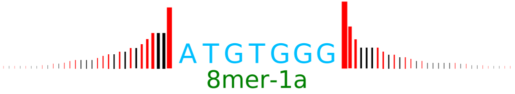

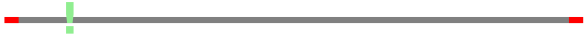

Supplement: Supplementary file 1 [file cells-10-01086-s001.zip › cells-1149161 3rd supplementary materials/Data Supplements/Data Supplement V.zip!/Data supplement 6-mRNAs targets of tsRNAs/tsRNA-Glu-TTC-010/Diagrams/tRF-Glu-TTC-010_vs_ENST00000343949(ZNF763).pdf]
